# Association of body mass index, metabolic health status and clinical outcomes in acute myocardial infarction patients: a national registry-based study

**DOI:** 10.3389/fcvm.2023.1142078

**Published:** 2023-06-26

**Authors:** Ching-Hui Sia, Junsuk Ko, Huili Zheng, Andrew Fu-Wah Ho, David Foo, Ling-Li Foo, Patrick Zhan-Yun Lim, Boon Wah Liew, Ping Chai, Tiong-Cheng Yeo, James W. L. Yip, Terrance Chua, Mark Yan-Yee Chan, Jack Wei Chieh Tan, Heerajnarain Bulluck, Derek J. Hausenloy

**Affiliations:** ^1^ Department of Cardiology, National University Heart Centre Singapore, Singapore; ^2^ Yong Loo Lin School of Medicine, National University of Singapore, Singapore; ^3^ MD Program, Duke-NUS Medical School, Singapore; ^4^ Health Promotion Board, National Registry of Diseases Office, Singapore, Singapore; ^5^ SingHealth Duke-NUS Emergency Medicine Academic Clinical Programme, Singapore; ^6^ National Heart Research Institute Singapore, National Heart Centre Singapore, Singapore; ^7^ Pre-hospital and Emergency Care Research Centre, Health Services and Systems Research, Duke-NUS Medical School; ^8^ Tan Tock Seng Hospital, Singapore, Singapore; ^9^ Khoo Teck Puat Hospital, Singapore, Singapore; ^10^ Changi General Hospital, Singapore; ^11^ Department of Cardiology, National Heart Centre Singapore, Singapore; ^12^ Leeds Teaching Hospital NHS Trust, Leeds, United Kingdom; ^13^ Cardiovascular & Metabolic Disorders Program, Duke-National University of Singapore Medical School, Singapore; ^14^ The Hatter Cardiovascular Institute, University College London, London, United Kingdom; ^15^ Cardiovascular Research Center, College of Medical and Health Sciences, Asia University, Taiwan

**Keywords:** metabolism, acute myocardial infarction, metabolically healthy obesity (MHO), cardiovascular risk, obesity paradox, AMI mortality, AMI prognosis, MHO paradox

## Abstract

**Introduction:**

Obesity is an important risk factor for acute myocardial infarction (AMI), but the interplay between metabolic health and obesity on AMI mortality has been controversial. In this study, we aimed to elucidate the risk of short- and long-term all-cause mortality by obesity and metabolic health in AMI patients using data from a multi-ethnic national AMI registry.

**Methods:**

A total of 73,382 AMI patients from the national Singapore Myocardial Infarction Registry (SMIR) were included. These patients were classified into four groups based on the presence or absence of metabolic diseases, diabetes mellitus, hyperlipidaemia, and hypertension, and obesity: (1) metabolically-healthy-normal-weight (MHN); (2) metabolically-healthy-obese (MHO); (3) metabolically-unhealthy-normal-weight (MUN); and (4) metabolically-unhealthy-obese (MUO).

**Results:**

MHO patients had reduced unadjusted risk of all-cause in-hospital, 30-day, 1-year, 2-year, and 5-year mortality following the initial MI event. However, after adjusting for potential confounders, the protective effect from MHO on post-AMI mortality was lost. Furthermore, there was no reduced risk of recurrent MI or stroke within 1-year from onset of AMI by the MHO status. However, the risk of 1-year mortality was higher in female and Malay AMI patients with MHO compared to MHN even after adjusting for confounders.

**Conclusion:**

In AMI patients with or without metabolic diseases, the presence of obesity did not affect mortality. The exception to this finding were female and Malay MHO who had worse long-term AMI mortality outcomes when compared to MHN suggesting that the presence of obesity in female and Malay patients may confer worsened outcomes.

## Introduction

Cardiovascular diseases (CVD) are the leading causes of mortality and morbidity worldwide accounting for 18.6 million deaths yearly (1). The prevalence of CVD significantly increased from 257 million in 1,990 to 523 million in 2019, adding substantially to the global healthcare burden (1). Obesity is a risk factor for the onset of various CVDs, including ischemic stroke (2), acute myocardial infarction (AMI) (3), and peripheral arterial disease (4). However, the association between obesity and poorer clinical outcomes in patients with CVD has been controversial.

Obesity is commonly defined as excessive fat accumulation. A body mass index (BMI) of 30 kg/m^2^ or greater is often used for diagnosis (5). However, based on BMI alone it is not enough to evaluate the effect of obesity on CVD prognosis as the phenotypes of obesity are heterogenous. Obesity status has been divided by some into four groups: (1) metabolically healthy obesity (MHO) (with no metabolic diseases such as hypertension, hyperlipidemia, and diabetes); (2) metabolically healthy normal weight (MHN) (no metabolic diseases and normal weight); (3) metabolically unhealthy obesity (MUO) (presence of metabolic diseases and obesity); and (4) metabolically unhealthy normal weight (MUN) (presence of metabolic diseases but normal weight) (6). Some studies have reported that MHO patients had a lower risk of all-cause mortality following MI events compared to MHN patients, suggesting that the presence of obesity in the absence of metabolic diseases may have a protective effect on post-AMI mortality (7–9). However, subsequent studies reported conflicting results, with MHO being associated with increased post-AMI mortality (10–12). These reports were cohort studies, where the exposure was obesity with or without metabolic diseases vis-a-vis normal weight with or without metabolic diseases, and the study population was followed up to see if MI and/or death occurred during the study period. To the best of our knowledge, no studies have investigated the association between obesity, metabolic health and clinical outcomes following AMI. Using data from a multi-ethnic national MI registry in this study, we stratified AMI patients by their obesity and metabolic health and investigated the effects of these factors on post-AMI mortality.

## Methods

### Data sources

This study utilized data from the Singapore Myocardial Infarction Registry (SMIR), a national registry maintained by the Ministry of Health and National Registry of Diseases Office. Written consent from study participants was waived by local institutional review board (SingHealth CIRB Reference No: 2016/2480) as de-identified data was used. This study was conducted in accordance with the Declaration of Helsinki. Anonymised individual-level data were accessed by the statistician for analysis, while only analysed and aggregated data were accessed by the co-authors for interpretation. The SMIR collects clinical data of all AMI patients in all hospitals in Singapore (13–17). Notification of AMI to the registry is mandated by law. The International Classification of Diseases, 9th Revision, Clinical Modification (ICD-9-CM) code 410 was used to identify AMI cases diagnosed prior to 2012, while ICD 10th Revision Australian Modification (ICD-10-AM) codes I21 and I22 were used for cases diagnosed from 2012 onwards. Patients' data were extracted from their medical records by the registry coordinators. The quality of the SMIR data is maintained by annual audits which inspect accuracy and inter-rater reliability, with outliers and illogical data flagged for review. The multinational monitoring of trends and determinants in cardiovascular disease criteria were used to define episodes (18).

Patients with AMI in 2008–2017 were matched with the death data (up till 31 July 2019) from the Registry of Births and Death for their survival status at discharge, 30 days and and 1 year after the onset of MI. Patients without the complete 2-year and 5-year follow-up duration due to the limited availability of death data were excluded, i.e., patients with AMI in 2008–2016 were included in the analysis for 2-year mortality, while patients with AMI in 2008–2013 were included in the analysis for 5-year mortality.

### Definition of obesity and metabolic health

In this study, a patient was deemed to be obese if the BMI ≥27.5 kg/m^2^, the cutoff value for Asian populations (19). Data on BMI was based on the weight measured during the admission for AMI and the latest measured height documented in the medical records. We did not assess abdominal obesity as data on waist circumference was not captured by the registry. A patient was deemed to be metabolically unhealthy if at least one of the three conditions (hypertension, diabetes, hyperlipidemia) was present based on medical history and diagnosis in the admission for AMI. Medical histories of hypertension, diabetes, and hyperlipidemia were based on past diagnoses and prescribed treatments. The diagnosis of hypertension was based on systolic blood pressure >140 mmHg or diastolic blood pressure >85 mmHg. The diagnosis of diabetes was based on fasting blood glucose ≥7.0 mmol/L or random glucose ≥11.1 mmol/L. The diagnosis of hyperlipidemia was based on total cholesterol >6.2 mmol/L, low-density lipoprotein (LDL) cholesterol >4.1 mmol/L, or triglyceride >1.7 mmol/L.

### Statistical analysis

The AMI patients were classified into four groups based on their obesity and metabolic health: metabolically healthy normal weight (MHN), metabolically healthy obese (MHO), metabolically unhealthy normal weight (MUN) and metabolically unhealthy obese (MUO). Demographic and clinical characteristics of the four groups of patients were presented using count with percentage for categorical variables and median with interquartile range for numeric variables. Chi-square test for categorical variables and Kruskal–Wallis rank test for numeric variables were used to compare the patients' characteristics across the four groups. Histograms were plotted to visually examine the age distribution of the AMI patients in each group. Survival time of all patients was calculated from the date of AMI onset to death or censor, whichever earlier. Cumulative mortality of the four groups of AMI patients were plotted using the Kaplan–Mier method to see if the mortality rate differ across the groups over the study period. Two short-term and three long-term mortality outcomes were analysed: death during hospitalisation (short-term), death within 30 days from onset of AMI (short-term), death within 1 year (long-term), death within 2 years (long-term) and death within 5 years (long-term). Restricted cubic splines were used to recode BMI and examine its association with mortality. Univariable cox regression with survival time as outcome and patient's group as predictor (model 1) were performed to compare the risk of death among MHO, MUN and MUO patients, relative to MHN patients. As metabolic health and body composition are known to differ by age and sex, multivariable cox regression including age and sex as covariates (model 2) were also done to compare the mortality risk across the groups. Furthermore, to account for other potential confounding variables captured by the registry, aside from age and sex, race, history of MI/PCI/CABG, smoking status, Killip class, haemoglobin, creatinine, MI type and in-hospital treatments were added into the multivariable cox regression (model 3). As part of sensitivity analysis, cox regression for survival time within 1, 2 and 5 years of AMI were performed among patients who survived beyond discharge and 30 days after AMI (i.e., excluding those who died during hospitalisation or within 30 days).

Similar cox regression were extended to subgroups of AMI patients (aged <60 years, aged ≥60 years, males, females, Chinese, Malays, Indians, never smokers, ex-smokers, current smokers) to see if the findings differ across the various subgroups.

All statistical analyses were performed using Stata (StataCorp. 2013. Stata Statistical Software: Release 13. College Station, TX: StataCorp LP). All statistical tests were 2-tailed, and results were deemed to be statistically significant if *p* < 0.05.

## Results

### Study population

**Figure 1:**
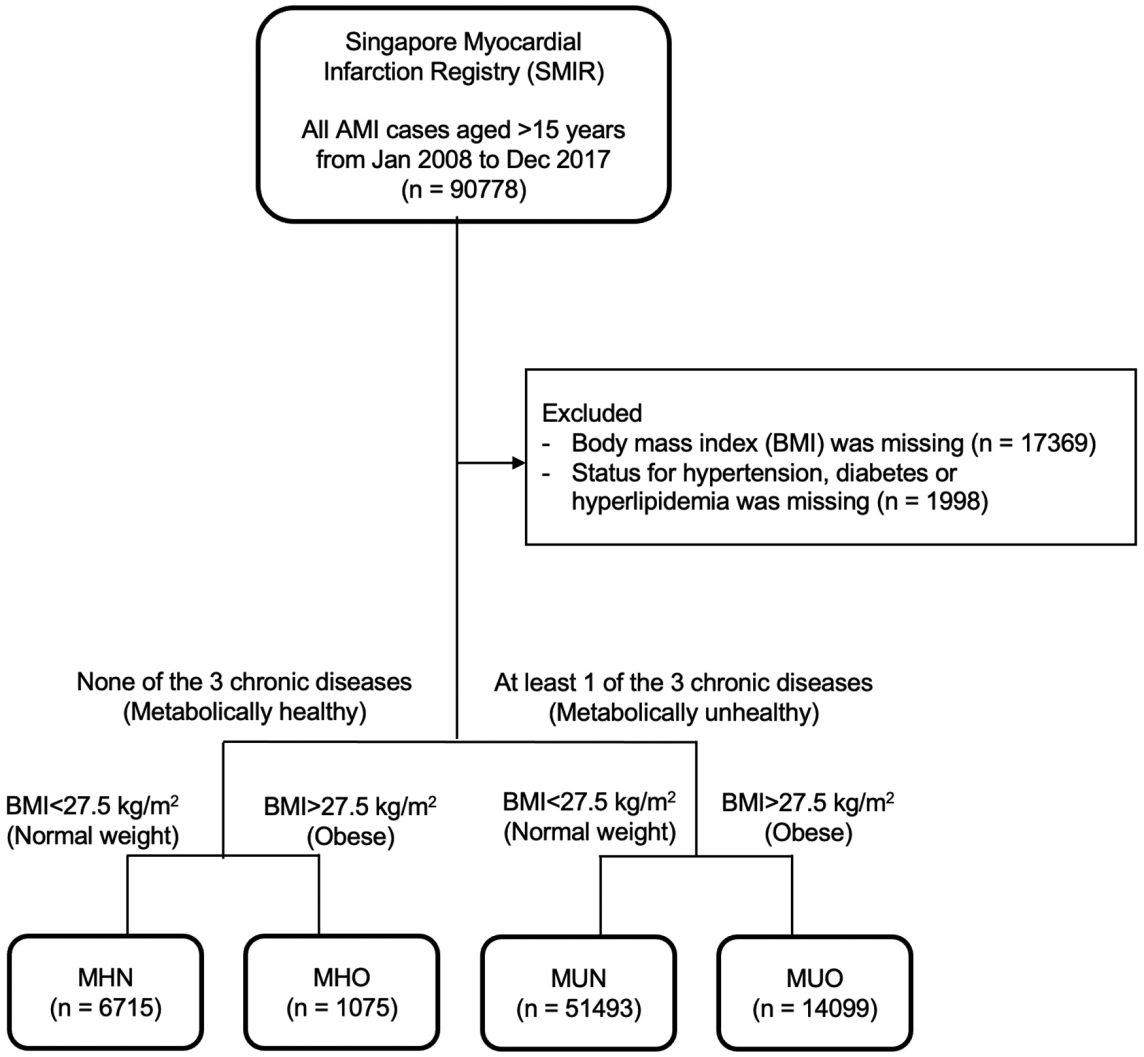
Flow chart of study population. 73,382 AMI patients were included in this study after excluding 17,396 patients with missing obesity and metabolic health status.

**Figure 2:**
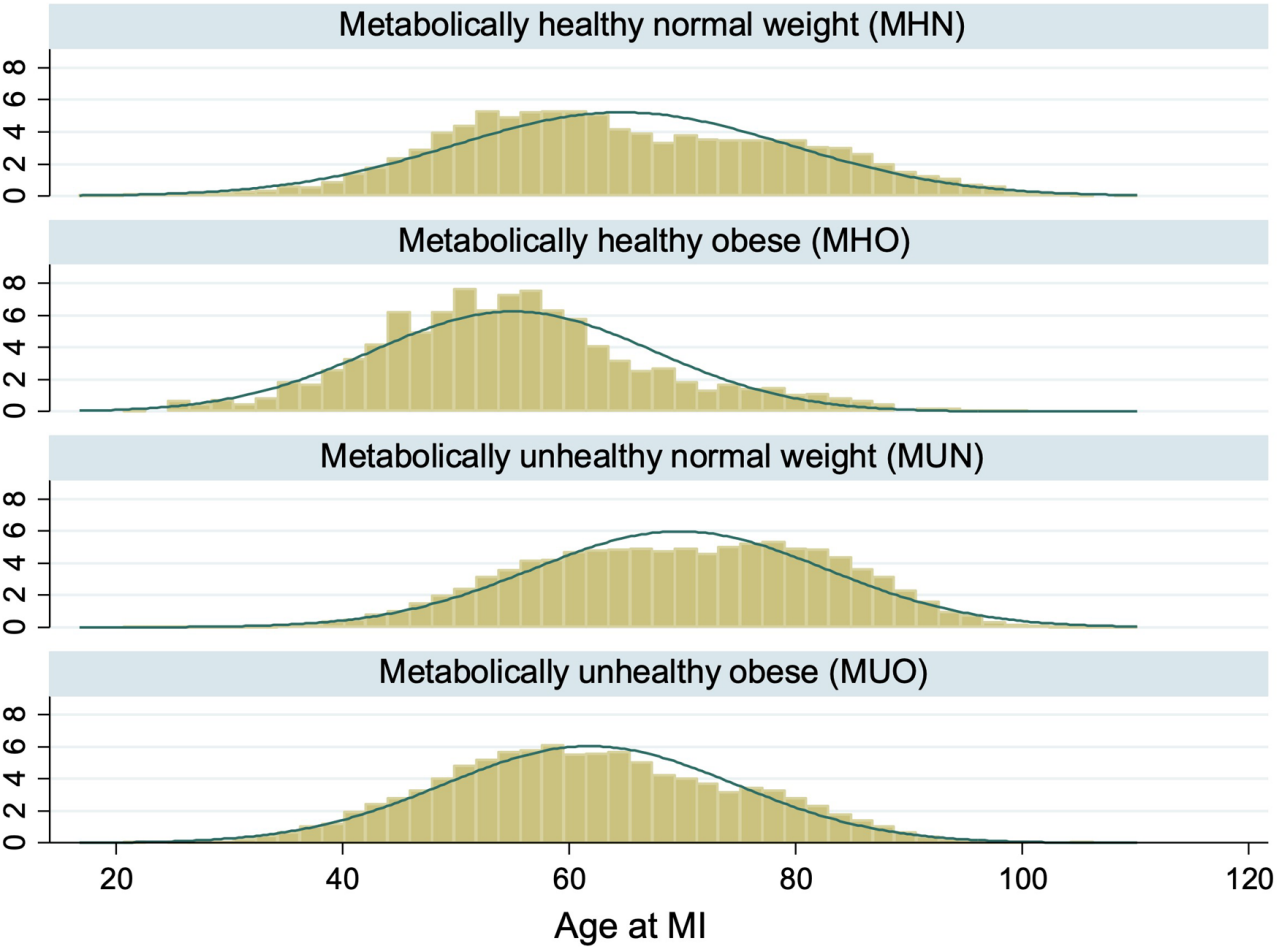
Distribution of age at AMI by obesity and metabolic health. The histograms showed that AMI patients who were obese tended to be younger than AMI patients who were non-obese regardless of metabolic health.

73,382 AMI patients were included in this study after excluding 17,396 patients with missing BMI and metabolic health status (Figure 1). There were 6,715 (9.2%) MHN, 1,075 (1.5%) MHO, 51,493 (70.2%) MUN and 14,099 (19.2%) MUO patients (Table 1). The median age of obese patients was younger and they generally had AMI at a younger age than non-obese patients, regardless of their metabolic health (Figure 2). The proportions of non-Chinese and those who received the evidence-based therapies during hospitalization were higher among obese patients compared to non-obese patients. The proportions of current smokers, Killip class I on admission and ST-segment elevation MI were higher among metabolically healthy patients than metabolically unhealthy patients. The median haemoglobin value on admission for AMI of the metabolically healthy patients was higher, but their median serum creatinine value was lower than metabolically unhealthy patients.

**Table 1:** Characteristics of AMI patients by obesity and metabolic health.

	Metabolically healthy		Metabolically unhealthy		
	Non-obese (MHN)	Obese (MHO)	Non-obese (MUN)	Obese (MUO)	
	*n* = 6,715	*n* = 1,075	*n* = 51,493	*n* = 14,099	*p*
**Demographics**					
Age in years, median (IQR)	63 (54–76)	54 (47–62)	70 (60–80)	61 (53–71)	<0.001
Male, *n* (%)	5,235 (78.0)	891 (82.9)	33,715 (65.5)	9,562 (67.8)	<0.001
**Race, *n* (%)**					
Chinese	4,869 (72.5)	584 (54.3)	35,276 (68.5)	7,054 (50.0)	<0.001
Malay	1,087 (16.2)	281 (26.1)	9,049 (17.6)	4,189 (29.7)	
Indian	648 (9.7)	175 (16.3)	6,497 (12.6)	2,607 (18.5)	
Others	111 (1.6)	35 (3.3)	671 (1.3)	249 (1.8)	
**Risk factors**					
Hypertension, *n* (%)	NA	NA	42,426 (82.4)	11,885 (84.3)	<0.001
Diabetes, *n* (%)	NA	NA	28,493 (55.3)	8,891 (63.1)	<0.001
Hyperlipidemia, *n* (%)	NA	NA	40,923 (79.5)	11,617 (82.4)	<0.001
History of MI/PCI/CABG, *n* (%)	529 (7.9)	70 (6.5)	18,698 (36.3)	4,649 (33.0)	<0.001
**Smoking status, *n* (%)**					
Current	2,867 (43.7)	525 (49.7)	11,842 (23.4)	3,819 (27.4)	<0.001
Former	1,055 (16.1)	156 (14.8)	11,327 (22.4)	2,716 (19.5)	
Never	2,646 (40.3)	375 (35.5)	27,498 (54.3)	7,397 (53.1)	
**Killip class on admission, *n* (%)**					
I	5,748 (85.7)	940 (87.4)	38,625 (75.1)	10,578 (75.1)	<0.001
II	409 (6.1)	61 (5.7)	6,698 (13.0)	1,855 (13.2)	
III	230 (3.4)	41 (3.8)	4,746 (9.2)	1,313 (9.3)	
IV	319 (4.8)	33 (3.1)	1,381 (2.7)	346 (2.5)	
**Lipid in mmol/L within 72 h from MI onset, median (IQR)**					
Total cholesterol	4.7 (4.0–5.3)	4.9 (4.3–5.4)	4.7 (3.8–5.8)	4.9 (4.0–6.0)	<0.001
Low-density lipoprotein cholesterol	3.1 (2.4–3.6)	3.2 (2.7–3.6)	2.9 (2.1–3.9)	3.1 (2.3–4.1)	<0.001
High-density lipoprotein cholesterol	1.0 (0.9–1.3)	0.9 (0.8–1.1)	1.1 (0.9–1.3)	1.0 (0.8–1.2)	<0.001
Triglyceride	1.2 (0.8–1.6)	1.5 (1.1–2.2)	1.3 (1.0–1.9)	1.6 (1.2–2.3)	<0.001
HbA1c in % on admission, median (IQR)	5.6 (5.4–5.9)	5.7 (5.5–6.0)	6.4 (5.7–7.8)	6.8 (5.9–8.3)	<0.001
Haemoglobin in g/dl on admission, median (IQR)	13.7 (11.9–15.1)	14.8 (13.6–15.8)	12.5 (10.6–14.3)	13.4 (11.4–15.1)	<0.001
Serum creatinine in µmol on admission, median (IQR)	87 (72–109)	88 (74–104)	106 (81–175)	102 (80–166)	<0.001
**Type of MI, *n* (%)**					
STEMI	2,715 (40.4)	541 (50.3)	12,537 (24.3)	3,882 (27.5)	<0.001
NSTEMI	3,410 (50.8)	468 (43.5)	34,700 (67.4)	9,331 (66.2)	
Unclassified	590 (8.8)	66 (6.1)	4,256 (8.3)	886 (6.3)	
**Treatment during hospitalization**					
PCI/CABG, *n* (%)	3,230 (48.1)	716 (66.6)	18,452 (35.8)	6,826 (48.4)	<0.001
Aspirin, *n* (%)	5,371 (80.0)	949 (88.3)	41,283 (80.2)	12,239 (86.8)	<0.001
Beta blocker, *n* (%)	4,484 (66.8)	838 (78.0)	39,700 (77.1)	11,620 (82.4)	<0.001
ACEI/ARB, *n* (%)	3,105 (46.2)	647 (60.2)	30,260 (58.8)	9,609 (68.2)	<0.001
Lipid lowering drug, *n* (%)	5,186 (77.2)	938 (87.3)	44,380 (86.2)	12,882 (91.4)	<0.001
P2Y12 inhibitor, *n* (%)	4,897 (72.9)	912 (84.8)	39,143 (76.0)	11,819 (83.8)	<0.001

ACEI/ARB, angiotensin converting enzyme inhibitor/angiotensin receptor blocker; CABG, coronary artery bypass grafting; IQR, interquartile range; MI, myocardial infarction; NA, not applicable; NSTEMI, non-ST-segment elevation myocardial infarction; PCI, percutaneous coronary intervention; STEMI, ST-segment elevation myocardial infarction.

**Figure 3:**
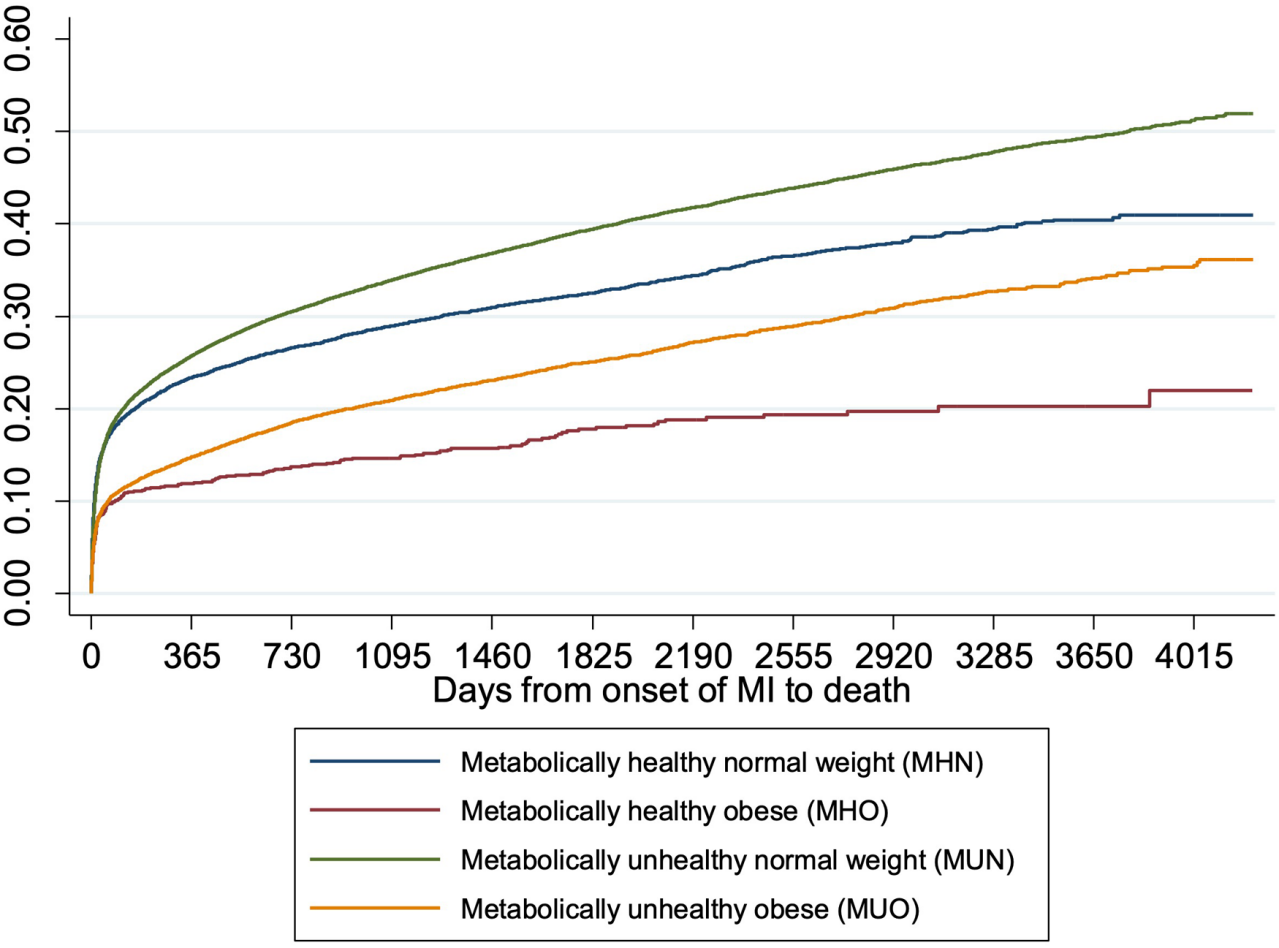
Cumulative incidence of death over time by obesity and metabolic health. The cumulative incidence curves showed that the unadjusted all-cause mortality was highest among MUN patients, followed by MHN, MUO and MHO patients.

**Figure 4:**
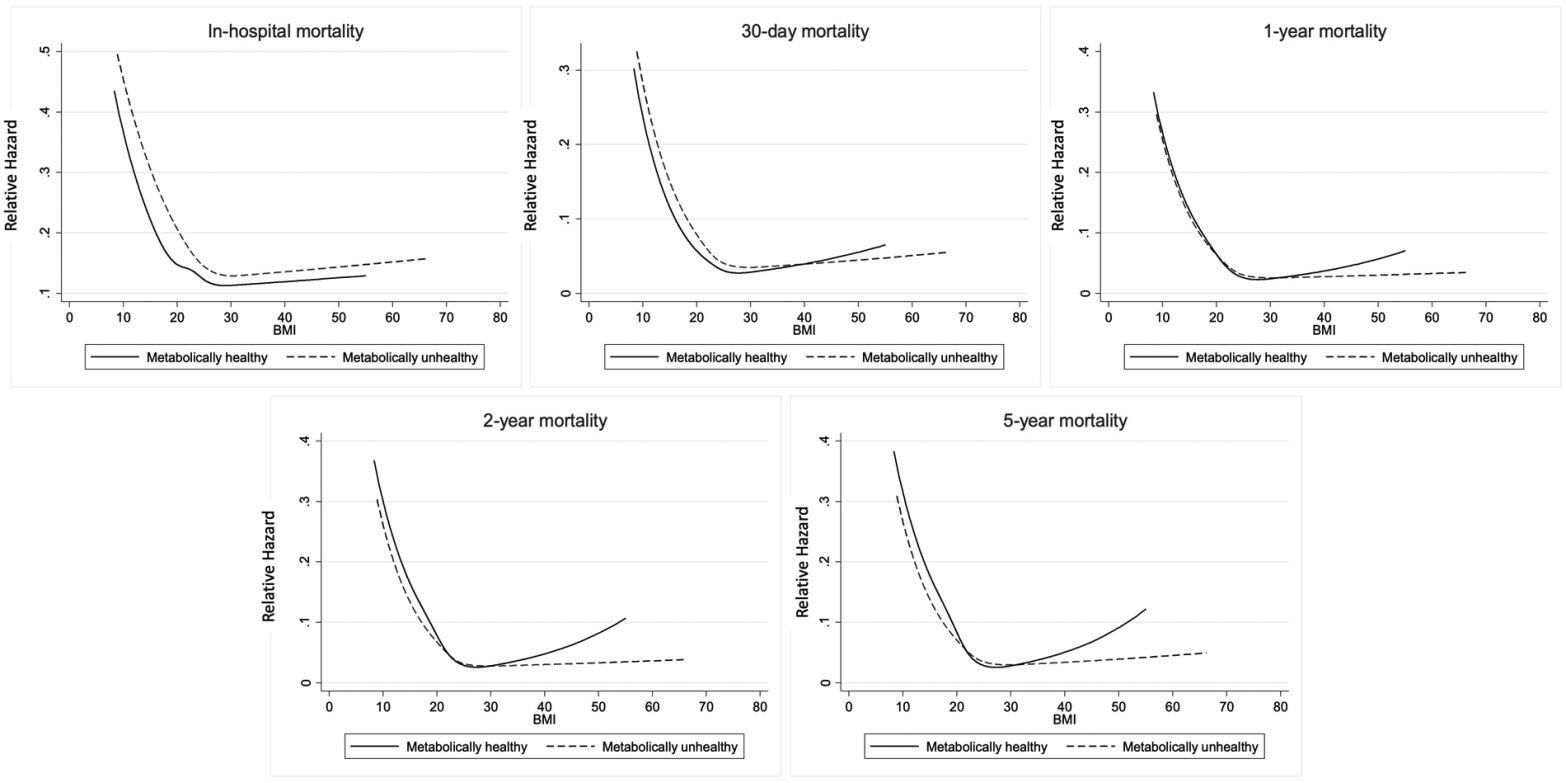
Mortality risk over BMI by metabolic health. The restricted cubic splines showed that mortality risk were lowest for patients with BMI in the healthy range regardless of metabolic health.

Plot of the cumulative mortality over time showed that the unadjusted all-cause mortality was highest among MUN patients, followed by MHN, MUO and MHO patients (Figure 3). Plots of the mortality risk over BMI showed that the mortality risk were lowest for patients with BMI in the healthy range regardless of metabolic health, and the mortality risk increased when BMI >25 kg/m^2^, especially for metabolically healthy patients (Figure 4).

### Short-term mortality

Compared to MHN patients, the unadjusted risk for in-hospital all-cause mortality was 23% (HR: 0.77, 95% CI: 0.62–0.96) lower among MHO patients, 14% (HR: 0.86, 95% CI: 0.80–0.92) lower among MUN patients, and 38% (HR: 0.62, 95% CI: 0.57–0.68) lower among MUO patients (Model 1, Table 2). However, after adjustment for age and sex, the risk among MHO patients (HR: 1.04, 95% CI: 0.84–1.30) was no longer significantly different from MHN patients. However, the MUN (HR: 0.78, 95% CI: 0.73–0.84) and MUO (HR: 0.70, 95% CI: 0.64–0.77) patients had still lower HR even after adjustment for age and sex than MHN patients (Model 2, Table 2). Of note, when adjusted for other potential confounding factors, including not only age and sex but also race, history of MI/PCI/CABG, smoking status, Killip class on admission, creatinine on admission, haemoglobin on admission, MI type, PCI/CABG during hospitalization, drugs (aspirin, P2Y12 inhibitor, beta blocker, ACEI/ARB, lipid lowering drug) given during hospitalization, the MUN and MUO patients had increased HRs suggesting that metabolic health, rather than obesity, was an independent predictor of in-hospital mortality (Model 3, Table 2).

**Table 2:** Risk of all-cause mortality in AMI patients by obesity and metabolic health.

	Metabolically healthy		Metabolically unhealthy	
	Non-obese (MHN)	Obese (MHO)	Non-obese (MUN)	Obese (MUO)
**In-hospital mortality**				
Model 1, HR (95% CI)	1.00 (ref)	0.77 (0.62–0.96)	0.86 (0.80–0.92)	0.62 (0.57–0.68)
Model 2, HR (95% CI)	1.00 (ref)	1.04 (0.84–1.30)	0.78 (0.73–0.84)	0.70 (0.64–0.77)
Model 3, HR (95% CI)	1.00 (ref)	1.04 (0.82–1.31)	1.29 (1.20–1.39)	1.26 (1.14–1.38)
**30-day mortality**				
Model 1, HR (95% CI)	1.00 (ref)	0.56 (0.45–0.70)	0.96 (0.90–1.03)	0.56 (0.52–0.61)
Model 2, HR (95% CI)	1.00 (ref)	0.94 (0.75–1.16)	0.79 (0.74–0.85)	0.67 (0.61–0.73)
Model 3, HR (95% CI)	1.00 (ref)	1.12 (0.89–1.40)	1.28 (1.19–1.38)	1.22 (1.11–1.34)
**1-year mortality**				
Model 1, HR (95% CI)	1.00 (ref)	0.48 (0.40–0.57)	1.10 (1.05–1.16)	0.59 (0.56–0.64)
Model 2, HR (95% CI)	1.00 (ref)	0.81 (0.67–0.97)	0.89 (0.84–0.94)	0.70 (0.66–0.75)
Model 3, HR (95% CI)	1.00 (ref)	0.97 (0.80–1.18)	1.19 (1.12–1.26)	1.01 (0.94–1.09)
**2-year mortality**				
Model 1, HR (95% CI)	1.00 (ref)	0.49 (0.41–0.59)	1.14 (1.08–1.20)	0.64 (0.60–0.68)
Model 2, HR (95% CI)	1.00 (ref)	0.84 (0.70–1.00)	0.92 (0.87–0.97)	0.75 (0.71–0.81)
Model 3, HR (95% CI)	1.00 (ref)	0.98 (0.81–1.19)	1.18 (1.11–1.24)	1.02 (0.95–1.09)
**5-year mortality**				
Model 1, HR (95% CI)	1.00 (ref)	0.51 (0.41–0.63)	1.20 (1.12–1.28)	0.69 (0.63–0.74)
Model 2, HR (95% CI)	1.00 (ref)	0.85 (0.68–1.06)	0.98 (0.92–1.05)	0.83 (0.77–0.90)
Model 3, HR (95% CI)	1.00 (ref)	0.98 (0.78–1.22)	1.13 (1.05–1.21)	0.99 (0.91–1.08)

CI, confidence interval; HR, hazard ratio; Model 1: Not adjusted for any covariate; Model 2: Adjusted for age and sex; Model 3: Adjusted for age, sex, race, history of MI/PCI/CABG, smoking status, Killip class on admission, creatinine on admission, haemoglobin on admission, MI type, PCI/CABG during hospitalization, drugs (aspirin, P2Y12 inhibitor, beta blocker, ACEI/ARB, lipid lowering drug) given during hospitalization.

Similarly, compared to MHN patients, the unadjusted risk for 30-day all-cause mortality was lower among MHO (HR: 0.56, 95% CI: 0.45–0.70) and MUO (HR: 0.56, 95% CI: 0.52–0.61) but not MUN patients (Model 1, Table 2). When adjusted for age and sex, the MUN and MUO but not MHO patients had lower HR (Model 2, Table 2, HR: 1.04, 95% CI: 0.84–1.30 for MHO, HR: 0.78, 95% CI: 0.73–0.84 for MUN, HR: 0.70, 95% CI: 0.64–0.77). However, the adjusted risk in model 3 was higher among MUN (HR: 1.28, 95% CI: 1.19–1.38) and MUO (HR: 1.22, 95% CI: 1.11–1.34) but not MHO patients (Table 2). Overall, the MHO status was not a protected factor consistently for both in-hospital and 30-day mortality.

### Long-term mortality

Similar results were observed for 1-year, 2-year and 5-year mortality. When compared to MHN patients, MHO and MUO patients had a lower unadjusted risk of mortality, while MUN patients had higher unadjusted and adjusted risk (Model 1, Table 2). The MHO, MUN, and MUO patients had lower HR for 1-year and 2-year mortality, but not MHO patients, when adjusted for age and sex (Model 2, Table 2). Similarly, the MUO group had lower 5-year mortality but not MHO patients. The adjusted HRs via model 3 suggested that the MUN status is an independent predictor of 1-year, 2-year and 5-year mortality, but not the MHO status.

When the patients who died during in-hospital and within 30 days were excluded, the MUO patients had lower 1, 2, and 5-year adjusted mortality but not the MHO patients compared to the MHN patients (Supplementary Table S1, model 3, HR: 0.73, 95% CI: 0.65–0.82 for 1-year, HR: 0.80, 95% CI: 0.72–0.89 for 2-year, and HR: 0.85, 95% CI: 0.76–0.96 for 5-year mortality). This result suggests that the MHO status is not a protective factor even when the patients with short-term mortality were excluded.

### Mortality by subgroups

Stratified adjusted analysis revealed that MHO was not associated with a reduced or increased risk of short-term and long-term mortality in all subgroups of AMI patients, including younger age (Supplementary Table S2), older age (Supplementary Table S3), male (Supplementary Table S4), Chinese ethnicity (Supplementary Table S6), Indian (Supplementary Table S8) except females (Supplementary Table S5) and Malay (Supplementary Table S7) in model 3. Intriguingly, among female AMI patients, MHO patients had a markedly elevated risk of 1-year (HR: 1.42, 95% CI: 1.06–1.90), 2-year (HR: 1.47, 95% CI: 1.08–2.01) and 5-year (HR: 1.49, 95% CI: 1.00–2.22) mortality even after accounting for other potential confounders (Supplementary Table S5). Similarly, Malay MHO patients had an increased risk of 5-year all cause mortality (HR: 1.58, 95% CI: 1.04–2.40) after adjustment.

Among never smokers, the MUN and MUO patients were associated with increased adjusted all-cause mortality at in-hospital, 30-day, 1-year, 2-year, and 50-year (Supplementary Table S9, in-hospital HR: 1.41, 95% CI: 1.27–1.56 and 1.45, 95%: 1.27–1.63 for MUN and MUO, 30-day HR: 1.42, 95% CI: 1.29–1.57 and 1.44, 95%: 1.28–1.63 for MUN and MUO, 1-year HR: 1.31, 95% CI: 1.21–1.41 and 1.14, 95%: 1.04–1.25 for MUN and MUO, 2-year HR: 1.30, 95% CI: 1.20–1.41 and 1.15, 95%: 1.05–1.27 for MUN and MUO, 5-year HR: 1.25, 95% CI: 1.13–1.38 and 1.15, 95%: 1.03–1.29 for MUN and MUO). Interestingly, the MUO patients who were ex-smokers or current smoker had lower adjusted 5-year mortality compared to the MHN patients who were ex-smokers (Supplementary Tables S10 and S11, HR: 0.83, 95% CI: 0.70–0.99 for ex-smokers and HR: 0.79, 95% CI: 0.66–0.95 for current smokers).

## Discussion

In this national registry-based study of AMI patients, we demonstrated that both short-term and long-term unadjusted all-cause mortality as well as the risk of recurrent MI within 1-year from onset of MI were lower in the MHO patients. However, the protective effect from the MHO status was lost when the potential confounding factors were adjusted for, suggesting that the protection from MHO on post-AMI mortality was a pseudo-paradox. Furthermore, we found that the MHO patients were associated with earlier onset of AMI and female MHO patients had an increased risk of 1-year, 2-year, and 5-year all-cause mortality even after adjustments. This collectively suggests that MHO does not show a beneficial effect, but rather this status is associated with a worse prognosis in certain group of AMI patients.

It is accepted that the MHO status is associated with an increased risk of adverse cardiovascular diseases, such as ischemic stroke, peripheral arterial disease, and early onset of AMI (20–24). However, whether the MHO status is associated with an increased morality for AMI has been controversial due to the conflicting results. Two recent studies using national registries independently demonstrated that the MHO status was associated with higher all-cause mortality (10) and CVD mortality (11) in the UK and Norway cohort respectively in addition to a recent study with meta-analysis study (12). On the other hand, another recent study in a Korean cohort (8) and a meta-analysis suggested (9) that the MHO status was associated with a lower all-cause mortality after AMI. These conflicting results are partially due to the lack of consensus over the definition of obesity in the field.

Although the World Health Organization (WHO) defined obesity as BMI ≥30 kg/m^2^, the diagnosis of obesity using BMI alone has been criticized as a higher BMI does not accurately reflect deposition of visceral fat, the key player of metabolic diseases (6, 25, 26). As visceral fat is more inflammatory and is associated with metabolic disorders than subcutaneous fat (6, 27, 28), it has been proposed that waist circumference or waist to hip ratio should be used to estimate the visceral fat (11). To the best of our knowledge, there is only one study which utilized waist circumference as a marker for abdominal obesity in addition to BMI for general obesity and demonstrated that there was no protection in terms of adjusted mortality by the MHO status (11). This method has not been widely used as waist circumference or waist-to-hip ratio is not available in many national CVD registries. Additionally, the cut-off for BMI to define metabolically healthy obesity has been controversial. Some studies used 25 as a cut-off for BMI to define overweight/obesity (8, 20) or used an ICD-10-AM code (E65) to define obesity (21). The inconsistent definition of obesity led to a wide range in the proportions of obese patients within the cohort, from 6.3% (29) to 41.27% (20) in recent studies. In this study, we used BMI ≥27.5 kg/m^2^ to define obesity as values above this cut-off have been associated with increased risk of CVD in Asian patients (19).

Due to the inconsistent definition of MHO in literature, the proportion of MHO patients has varied from 7.8% (11) to 16.2% (8). This difference could be in part due to the presence of undiagnosed cases within the cohort and the quality issues of the dataset. As such, we utilized data from a national registry which undergoes annual audits and uses not only the medical records but also the laboratory values obtained at admission to determine the presence of metabolic diseases. In fact, only 1.46% AMI patients were MHO in this cohort compared to the higher numbers in previous studies, suggesting that if the definition of metabolic health is not strict there could be inappropriate classification of patients. Nevertheless, our cohort differed from the populations of other studies and our results need to be further validated in other cohorts too.

Intriguingly, the sub-group analysis revealed that the female MHO patients were associated with increased all-cause 1-year, 2-year, and 5-year mortality following AMI (Table 3). A previous study demonstrated that female MHO patients have a higher CVD disease risk compared to MHN patients (HR: 4.65, 95% CI: 1.61–13.44) (20). In contrast, another recent study reported that the female MHO patients had a lower risk of CVD risks compared to their male counterparts (21). These results suggest that sex may be an important effect modifier, but there has not been any mortality data available prior to this study. Our data suggest that the female MHO patients have a poorer all-cause mortality following AMI. In addition to the poorer mortality by the MHO status, MHO is associated with an earlier onset of MI (30). Consistent with this report, our data suggests that the MHO patients who had an AMI event tended to be younger than the MHN patients. Collectively, MHO status was associated with an increased risk of AMI events and as well as early onset in young patients. Furthermore, a certain group of MHO patients may have a worse prognosis following the AMI events.

## Strengths and limitations

This study had several strengths. Firstly, the patient population was obtained from a national MI registry, where the data quality is maintained via regular audit and comprehensive case capture is ensured via mandatory notification. The MI data was complemented by death data from the national death registry to track survival outcomes without losing follow-ups. Moreover, this study looked at not only short-term but also long-term mortality among AMI patients, including further analyses by subgroups of patients and analysing BMI in both numeric and binary form.

Nonetheless, this study had several limitations. As this was a retrospective study, causality could not be determined in this study. Obesity status was defined as BMI ≥27.5 kg/m^2^ in this study, and neither waist-to-hip ratio nor waist circumference were available to better define obesity (31). There is emerging evidence that low BMI is associated with increased mortalities after AMI events (32). Nonetheless, the lower cut-off for normal BMI was not set for the analyses in this study due to the controversies over the best cut-off to use. Furthermore, this study only looked at BMI measured at one time point (during the admission for AMI), which did not reflect the change of BMI over time or at the point of death. However, temporal changes in BMI and its influence on outcomes can be the focus of future studies.

## Summary and conclusions

In this national registry of AMI patients, the presence of metabolic risk factors (DM, hyperlipidaemia, or hypertension) increased the risk of both short- and long-term AMI mortality, recurrent MI and stroke in both non-obese and obese patients. This effect was most pronounced in the Malay and Indian AMI patients when compared to the Chinese AMI patients. In AMI patients with or without metabolic risk factors (DM, hyperlipidaemia, or hypertension), the presence of obesity did not affect short- or long-term mortality. The exception to this finding were female MHO patients who had worse 1-, 2- and 5-AMI mortality outcomes when compared to MHN patients suggesting that in female AMI patients the presence of obesity conferred worsened outcomes.

## Data Availability

The raw data supporting the conclusions of this article will be made available by the authors, without undue reservation.
