# Supplementary material for: Association of body mass index, metabolic health status and clinical outcomes in acute myocardial infarction patients: a national registry-based study
Source: Front Cardiovasc Med. 2023 Jun 26;10:1142078. doi: 10.3389/fcvm.2023.1142078 (PMC10331723; doi:10.3389/fcvm.2023.1142078)
Supplement: Supplementary file 1 [file Table1.docx]

Supplementary Table 1. Risk of all-cause mortality in AMI patients by obesity and metabolic health among those who survived beyond discharge and 30 days after AMI

|  | **Metabolically healthy** | | **Metabolically unhealthy** | |
| --- | --- | --- | --- | --- |
|  | **Non-obese (MHN)** | **Obese**  **(MHO)** | **Non-obese (MUN)** | **Obese**  **(MUO)** |
| **1-year mortality** |  |  |  |  |
| Model 1, HR (95% CI) | 1.00 (ref) | 0.33 (0.23-0.48) | 1.34 (1.22-1.47) | 0.63 (0.57-0.71) |
| Model 2, HR (95% CI) | 1.00 (ref) | 0.58 (0.40-0.83) | 1.05 (0.96-1.15) | 0.74 (0.66-0.83) |
| Model 3, HR (95% CI) | 1.00 (ref) | 0.69 (0.47-1.00) | 1.00 (0.91-1.10) | 0.73 (0.65-0.82) |
| **2-year mortality** |  |  |  |  |
| Model 1, HR (95% CI) | 1.00 (ref) | 0.39 (0.29-0.53) | 1.36 (1.25-1.48) | 0.72 (0.66-0.80) |
| Model 2, HR (95% CI) | 1.00 (ref) | 0.68 (0.50-0.92) | 1.07 (0.99-1.17) | 0.85 (0.76-0.93) |
| Model 3, HR (95% CI) | 1.00 (ref) | 0.78 (0.57-1.07) | 1.00 (0.91-1.09) | 0.80 (0.72-0.89) |
| **5-year mortality** |  |  |  |  |
| Model 1, HR (95% CI) | 1.00 (ref) | 0.40 (0.29-0.56) | 1.40 (1.28-1.53) | 0.80 (0.72-0.89) |
| Model 2, HR (95% CI) | 1.00 (ref) | 0.67 (0.48-0.93) | 1.11 (1.02-1.22) | 0.96 (0.86-1.06) |
| Model 3, HR (95% CI) | 1.00 (ref) | 0.78 (0.56-1.08) | 0.99 (0.90-1.09) | 0.85 (0.76-0.96) |

Abbreviations: CI, confidence interval; HR, hazard ratio

Model 1: Not adjusted for any covariate

Model 2: Adjusted for age and sex

Model 3: Adjusted for age, sex, race, history of MI/PCI/CABG, smoking status, Killip class on admission, creatinine on admission, haemoglobin on admission, MI type, PCI/CABG during hospitalization, drugs (aspirin, P2Y12 inhibitor, beta blocker, ACEI/ARB, lipid lowering drug) given during hospitalization

Supplementary Table 2. Risk of all-cause mortality in AMI patients aged <60 years by obesity and metabolic health

|  | **Metabolically healthy** | | **Metabolically unhealthy** | |
| --- | --- | --- | --- | --- |
|  | **Non-obese (MHN)** | **Obese**  **(MHO)** | **Non-obese (MUN)** | **Obese**  **(MUO)** |
| **In-hospital mortality** |  |  |  |  |
| Model 1, HR (95% CI) | 1.00 (ref) | 0.91 (0.63-1.30) | 0.69 (0.58-0.81) | 0.55 (0.45-0.67) |
| Model 2, HR (95% CI) | 1.00 (ref) | 0.88 (0.61-1.25) | 0.67 (0.57-0.79) | 0.53 (0.44-0.64) |
| Model 3, HR (95% CI) | 1.00 (ref) | 0.92 (0.62-1.35) | 1.46 (1.21-1.76) | 1.28 (1.03-1.59) |
| **30-day mortality** |  |  |  |  |
| Model 1, HR (95% CI) | 1.00 (ref) | 0.74 (0.52-1.04) | 0.76 (0.65-0.90) | 0.54 (0.44-0.65) |
| Model 2, HR (95% CI) | 1.00 (ref) | 0.73 (0.52-1.03) | 0.73 (0.62-0.85) | 0.50 (0.41-0.60) |
| Model 3, HR (95% CI) | 1.00 (ref) | 1.00 (0.69-1.45) | 1.41 (1.18-1.69) | 1.15 (0.93-1.42) |
| **1-year mortality** |  |  |  |  |
| Model 1, HR (95% CI) | 1.00 (ref) | 0.71 (0.53-0.95) | 0.95 (0.84-1.09) | 0.71 (0.61-0.82) |
| Model 2, HR (95% CI) | 1.00 (ref) | 0.71 (0.52-0.94) | 0.91 (0.80-1.04) | 0.66 (0.57-0.77) |
| Model 3, HR (95% CI) | 1.00 (ref) | 0.92 (0.67-1.25) | 1.26 (1.09-1.46) | 1.06 (0.90-1.25) |
| **2-year mortality** |  |  |  |  |
| Model 1, HR (95% CI) | 1.00 (ref) | 0.76 (0.56-1.02) | 1.07 (0.94-1.23) | 0.84 (0.72-0.97) |
| Model 2, HR (95% CI) | 1.00 (ref) | 0.76 (0.56-1.02) | 1.02 (0.89-1.17) | 0.78 (0.67-0.91) |
| Model 3, HR (95% CI) | 1.00 (ref) | 0.94 (0.69-1.28) | 1.32 (1.14-1.54) | 1.14 (0.97-1.35) |
| **5-year mortality** |  |  |  |  |
| Model 1, HR (95% CI) | 1.00 (ref) | 0.74 (0.50-1.09) | 1.35 (1.14-1.59) | 1.07 (0.89-1.29) |
| Model 2, HR (95% CI) | 1.00 (ref) | 0.74 (0.50-1.09) | 1.29 (1.09-1.52) | 1.01 (0.84-1.21) |
| Model 3, HR (95% CI) | 1.00 (ref) | 0.83 (0.55-1.25) | 1.36 (1.13-1.63) | 1.12 (0.92-1.37) |

Abbreviations: CI, confidence interval; HR, hazard ratio

Model 1: Not adjusted for any covariate

Model 2: Adjusted for sex

Model 3: Adjusted for sex, race, history of MI/PCI/CABG, smoking status, Killip class on admission, creatinine on admission, haemoglobin on admission, MI type, PCI/CABG during hospitalization, drugs (aspirin, P2Y12 inhibitor, beta blocker, ACEI/ARB, lipid lowering drug) given during hospitalization

Supplementary Table 3. Risk of all-cause mortality in AMI patients aged ≥60 years by obesity and metabolic health

|  | **Metabolically healthy** | | **Metabolically unhealthy** | |
| --- | --- | --- | --- | --- |
|  | **Non-obese (MHN)** | **Obese**  **(MHO)** | **Non-obese (MUN)** | **Obese**  **(MUO)** |
| **In-hospital mortality** |  |  |  |  |
| Model 1, HR (95% CI) | 1.00 (ref) | 0.95 (0.71-1.26) | 0.81 (0.75-0.88) | 0.67 (0.60-0.73) |
| Model 2, HR (95% CI) | 1.00 (ref) | 0.94 (0.71-1.25) | 0.80 (0.74-0.87) | 0.65 (0.59-0.72) |
| Model 3, HR (95% CI) | 1.00 (ref) | 1.11 (0.82-1.50) | 1.30 (1.20-1.42) | 1.25 (1.13-1.39) |
| **30-day mortality** |  |  |  |  |
| Model 1, HR (95% CI) | 1.00 (ref) | 0.83 (0.62-1.10) | 0.83 (0.77-0.89) | 0.60 (0.55-0.66) |
| Model 2, HR (95% CI) | 1.00 (ref) | 0.83 (0.63-1.10) | 0.80 (0.74-0.86) | 0.58 (0.53-0.64) |
| Model 3, HR (95% CI) | 1.00 (ref) | 1.18 (0.88-1.59) | 1.31 (1.21-1.42) | 1.22 (1.10-1.36) |
| **1-year mortality** |  |  |  |  |
| Model 1, HR (95% CI) | 1.00 (ref) | 0.70 (0.55-0.88) | 0.91 (0.86-0.97) | 0.60 (0.56-0.64) |
| Model 2, HR (95% CI) | 1.00 (ref) | 0.70 (0.55-0.88) | 0.88 (0.83-0.94) | 0.57 (0.53-0.62) |
| Model 3, HR (95% CI) | 1.00 (ref) | 1.00 (0.78-1.27) | 1.22 (1.15-1.30) | 0.97 (0.89-1.05) |
| **2-year mortality** |  |  |  |  |
| Model 1, HR (95% CI) | 1.00 (ref) | 0.70 (0.56-0.89) | 0.92 (0.87-0.97) | 0.63 (0.58-0.67) |
| Model 2, HR (95% CI) | 1.00 (ref) | 0.71 (0.56-0.90) | 0.89 (0.84-0.94) | 0.60 (0.56-0.65) |
| Model 3, HR (95% CI) | 1.00 (ref) | 1.02 (0.80-1.30) | 1.18 (1.11-1.26) | 0.94 (0.87-1.02) |
| **5-year mortality** |  |  |  |  |
| Model 1, HR (95% CI) | 1.00 (ref) | 0.80 (0.61-1.05) | 0.92 (0.86-0.99) | 0.65 (0.59-0.70) |
| Model 2, HR (95% CI) | 1.00 (ref) | 0.81 (0.62-1.06) | 0.89 (0.83-0.96) | 0.62 (0.57-0.68) |
| Model 3, HR (95% CI) | 1.00 (ref) | 1.12 (0.85-1.48) | 1.08 (1.00-1.16) | 0.87 (0.79-0.95) |

Abbreviations: CI, confidence interval; HR, hazard ratio

Model 1: Not adjusted for any covariate

Model 2: Adjusted for sex

Model 3: Adjusted for sex, race, history of MI/PCI/CABG, smoking status, Killip class on admission, creatinine on admission, haemoglobin on admission, MI type, PCI/CABG during hospitalization, drugs (aspirin, P2Y12 inhibitor, beta blocker, ACEI/ARB, lipid lowering drug) given during hospitalization

Supplementary Table 4. Risk of all-cause mortality in male AMI patients by obesity and metabolic health

|  | **Metabolically healthy** | | **Metabolically unhealthy** | |
| --- | --- | --- | --- | --- |
|  | **Non-obese (MHN)** | **Obese**  **(MHO)** | **Non-obese (MUN)** | **Obese**  **(MUO)** |
| **In-hospital mortality** |  |  |  |  |
| Model 1, HR (95% CI) | 1.00 (ref) | 0.65 (0.49-0.88) | 0.85 (0.78-0.92) | 0.56 (0.50-0.63) |
| Model 2, HR (95% CI) | 1.00 (ref) | 0.92 (0.68-1.24) | 0.77 (0.70-0.84) | 0.67 (0.60-0.75) |
| Model 3, HR (95% CI) | 1.00 (ref) | 0.91 (0.66-1.24) | 1.23 (1.12-1.35) | 1.11 (0.98-1.26) |
| **30-day mortality** |  |  |  |  |
| Model 1, HR (95% CI) | 1.00 (ref) | 0.47 (0.36-0.62) | 0.96 (0.89-1.05) | 0.49 (0.43-0.54) |
| Model 2, HR (95% CI) | 1.00 (ref) | 0.80 (0.60-1.06) | 0.79 (0.72-0.86) | 0.62 (0.55-0.69) |
| Model 3, HR (95% CI) | 1.00 (ref) | 0.99 (0.74-1.33) | 1.20 (1.10-1.31) | 1.04 (0.92-1.18) |
| **1-year mortality** |  |  |  |  |
| Model 1, HR (95% CI) | 1.00 (ref) | 0.38 (0.30-0.48) | 1.12 (1.05-1.20) | 0.54 (0.50-0.59) |
| Model 2, HR (95% CI) | 1.00 (ref) | 0.64 (0.50-0.82) | 0.90 (0.84-0.96) | 0.69 (0.63-0.75) |
| Model 3, HR (95% CI) | 1.00 (ref) | 0.78 (0.61-1.00) | 1.13 (1.05-1.21) | 0.92 (0.84-1.01) |
| **2-year mortality** |  |  |  |  |
| Model 1, HR (95% CI) | 1.00 (ref) | 0.42 (0.33-0.52) | 1.15 (1.08-1.23) | 0.58 (0.54-0.64) |
| Model 2, HR (95% CI) | 1.00 (ref) | 0.70 (0.55-0.88) | 0.93 (0.87-0.99) | 0.74 (0.68-0.81) |
| Model 3, HR (95% CI) | 1.00 (ref) | 0.81 (0.64-1.03) | 1.10 (1.02-1.18) | 0.92 (0.84-1.01) |
| **5-year mortality** |  |  |  |  |
| Model 1, HR (95% CI) | 1.00 (ref) | 0.46 (0.35-0.61) | 1.23 (1.14-1.33) | 0.65 (0.59-0.72) |
| Model 2, HR (95% CI) | 1.00 (ref) | 0.74 (0.56-0.97) | 1.02 (0.94-1.11) | 0.85 (0.77-0.94) |
| Model 3, HR (95% CI) | 1.00 (ref) | 0.83 (0.63-1.10) | 1.07 (0.98-1.16) | 0.91 (0.82-1.01) |

Abbreviations: CI, confidence interval; HR, hazard ratio

Model 1: Not adjusted for any covariate

Model 2: Adjusted for age

Model 3: Adjusted for age, race, history of MI/PCI/CABG, smoking status, Killip class on admission, creatinine on admission, haemoglobin on admission, MI type, PCI/CABG during hospitalization, drugs (aspirin, P2Y12 inhibitor, beta blocker, ACEI/ARB, lipid lowering drug) given during hospitalization

Supplementary Table 5. Risk of all-cause mortality in female AMI patients by obesity and metabolic health

|  | **Metabolically healthy** | | **Metabolically unhealthy** | |
| --- | --- | --- | --- | --- |
|  | **Non-obese (MHN)** | **Obese**  **(MHO)** | **Non-obese (MUN)** | **Obese**  **(MUO)** |
| **In-hospital mortality** |  |  |  |  |
| Model 1, HR (95% CI) | 1.00 (ref) | 1.04 (0.75-1.45) | 0.81 (0.72-0.91) | 0.65 (0.56-0.75) |
| Model 2, HR (95% CI) | 1.00 (ref) | 1.31 (0.94-1.83) | 0.79 (0.70-0.89) | 0.73 (0.64-0.84) |
| Model 3, HR (95% CI) | 1.00 (ref) | 1.24 (0.87-1.78) | 1.41 (1.24-1.60) | 1.49 (1.28-1.73) |
| **30-day mortality** |  |  |  |  |
| Model 1, HR (95% CI) | 1.00 (ref) | 0.89 (0.63-1.25) | 0.79 (0.70-0.89) | 0.56 (0.49-0.65) |
| Model 2, HR (95% CI) | 1.00 (ref) | 1.33 (0.95-1.87) | 0.76 (0.68-0.86) | 0.69 (0.60-0.79) |
| Model 3, HR (95% CI) | 1.00 (ref) | 1.39 (0.96-2.00) | 1.44 (1.28-1.63) | 1.52 (1.31-1.76) |
| **1-year mortality** |  |  |  |  |
| Model 1, HR (95% CI) | 1.00 (ref) | 0.83 (0.63-1.09) | 0.86 (0.78-0.94) | 0.55 (0.49-0.61) |
| Model 2, HR (95% CI) | 1.00 (ref) | 1.27 (0.97-1.68) | 0.83 (0.76-0.90) | 0.67 (0.61-0.75) |
| Model 3, HR (95% CI) | 1.00 (ref) | 1.42 (1.06-1.90) | 1.30 (1.18-1.43) | 1.16 (1.04-1.30) |
| **2-year mortality** |  |  |  |  |
| Model 1, HR (95% CI) | 1.00 (ref) | 0.79 (0.59-1.06) | 0.89 (0.81-0.98) | 0.58 (0.52-0.65) |
| Model 2, HR (95% CI) | 1.00 (ref) | 1.29 (0.97-1.73) | 0.86 (0.79-0.94) | 0.72 (0.65-0.81) |
| Model 3, HR (95% CI) | 1.00 (ref) | 1.47 (1.08-2.01) | 1.31 (1.19-1.45) | 1.19 (1.06-1.33) |
| **5-year mortality** |  |  |  |  |
| Model 1, HR (95% CI) | 1.00 (ref) | 0.77 (0.53-1.11) | 0.88 (0.79-0.99) | 0.58 (0.51-0.66) |
| Model 2, HR (95% CI) | 1.00 (ref) | 1.31 (0.90-1.90) | 0.86 (0.77-0.97) | 0.74 (0.65-0.85) |
| Model 3, HR (95% CI) | 1.00 (ref) | 1.49 (1.00-2.22) | 1.24 (1.10-1.40) | 1.15 (1.00-1.32) |

Abbreviations: CI, confidence interval; HR, hazard ratio

Model 1: Not adjusted for any covariate

Model 2: Adjusted for age

Model 3: Adjusted for age, race, history of MI/PCI/CABG, smoking status, Killip class on admission, creatinine on admission, haemoglobin on admission, MI type, PCI/CABG during hospitalization, drugs (aspirin, P2Y12 inhibitor, beta blocker, ACEI/ARB, lipid lowering drug) given during hospitalization

Supplementary Table 6. Risk of all-cause mortality in Chinese AMI patients by obesity and metabolic health

|  | **Metabolically healthy** | | **Metabolically unhealthy** | |
| --- | --- | --- | --- | --- |
|  | **Non-obese (MHN)** | **Obese**  **(MHO)** | **Non-obese (MUN)** | **Obese**  **(MUO)** |
| **In-hospital mortality** |  |  |  |  |
| Model 1, HR (95% CI) | 1.00 (ref) | 0.70 (0.52-0.95) | 0.87 (0.80-0.94) | 0.65 (0.59-0.73) |
| Model 2, HR (95% CI) | 1.00 (ref) | 0.94 (0.70-1.27) | 0.78 (0.72-0.84) | 0.72 (0.65-0.80) |
| Model 3, HR (95% CI) | 1.00 (ref) | 1.01 (0.73-1.38) | 1.27 (1.16-1.38) | 1.27 (1.14-1.43) |
| **30-day mortality** |  |  |  |  |
| Model 1, HR (95% CI) | 1.00 (ref) | 0.54 (0.41-0.72) | 0.98 (0.90-1.05) | 0.60 (0.54-0.67) |
| Model 2, HR (95% CI) | 1.00 (ref) | 0.88 (0.66-1.18) | 0.78 (0.72-0.85) | 0.69 (0.62-0.76) |
| Model 3, HR (95% CI) | 1.00 (ref) | 1.04 (0.76-1.41) | 1.25 (1.15-1.36) | 1.26 (1.13-1.41) |
| **1-year mortality** |  |  |  |  |
| Model 1, HR (95% CI) | 1.00 (ref) | 0.46 (0.36-0.58) | 1.10 (1.03-1.17) | 0.60 (0.56-0.65) |
| Model 2, HR (95% CI) | 1.00 (ref) | 0.76 (0.60-0.96) | 0.86 (0.81-0.92) | 0.69 (0.63-0.75) |
| Model 3, HR (95% CI) | 1.00 (ref) | 0.88 (0.68-1.14) | 1.14 (1.07-1.22) | 1.01 (0.92-1.10) |
| **2-year mortality** |  |  |  |  |
| Model 1, HR (95% CI) | 1.00 (ref) | 0.45 (0.35-0.57) | 1.13 (1.07-1.21) | 0.65 (0.60-0.71) |
| Model 2, HR (95% CI) | 1.00 (ref) | 0.76 (0.59-0.97) | 0.89 (0.84-0.95) | 0.75 (0.69-0.81) |
| Model 3, HR (95% CI) | 1.00 (ref) | 0.84 (0.65-1.08) | 1.12 (1.05-1.20) | 1.02 (0.94-1.12) |
| **5-year mortality** |  |  |  |  |
| Model 1, HR (95% CI) | 1.00 (ref) | 0.44 (0.33-0.60) | 1.16 (1.08-1.25) | 0.68 (0.62-0.75) |
| Model 2, HR (95% CI) | 1.00 (ref) | 0.73 (0.54-0.98) | 0.92 (0.85-0.99) | 0.81 (0.73-0.89) |
| Model 3, HR (95% CI) | 1.00 (ref) | 0.77 (0.56-1.05) | 1.04 (0.96-1.12) | 0.98 (0.89-1.09) |

Abbreviations: CI, confidence interval; HR, hazard ratio

Model 1: Not adjusted for any covariate

Model 2: Adjusted for age and sex

Model 3: Adjusted for age, sex, race, history of MI/PCI/CABG, smoking status, Killip class on admission, creatinine on admission, haemoglobin on admission, MI type, PCI/CABG during hospitalization, drugs (aspirin, P2Y12 inhibitor, beta blocker, ACEI/ARB, lipid lowering drug) given during hospitalization

Supplementary Table 7. Risk of all-cause mortality in Malay AMI patients by obesity and metabolic health

|  | **Metabolically healthy** | | **Metabolically unhealthy** | |
| --- | --- | --- | --- | --- |
|  | **Non-obese (MHN)** | **Obese**  **(MHO)** | **Non-obese (MUN)** | **Obese**  **(MUO)** |
| **In-hospital mortality** |  |  |  |  |
| Model 1, HR (95% CI) | 1.00 (ref) | 1.07 (0.70-1.62) | 0.86 (0.71-1.04) | 0.62 (0.50-0.76) |
| Model 2, HR (95% CI) | 1.00 (ref) | 1.31 (0.86-1.99) | 0.81 (0.67-0.98) | 0.68 (0.55-0.84) |
| Model 3, HR (95% CI) | 1.00 (ref) | 1.26 (0.81-1.95) | 1.40 (1.14-1.72) | 1.34 (1.06-1.69) |
| **30-day mortality** |  |  |  |  |
| Model 1, HR (95% CI) | 1.00 (ref) | 0.70 (0.46-1.06) | 0.96 (0.81-1.14) | 0.57 (0.47-0.70) |
| Model 2, HR (95% CI) | 1.00 (ref) | 1.04 (0.69-1.59) | 0.82 (0.69-0.98) | 0.64 (0.53-0.79) |
| Model 3, HR (95% CI) | 1.00 (ref) | 1.35 (0.87-2.08) | 1.37 (1.13-1.65) | 1.21 (0.97-1.50) |
| **1-year mortality** |  |  |  |  |
| Model 1, HR (95% CI) | 1.00 (ref) | 0.59 (0.42-0.84) | 1.14 (1.00-1.31) | 0.65 (0.56-0.76) |
| Model 2, HR (95% CI) | 1.00 (ref) | 0.90 (0.64-1.28) | 0.97 (0.85-1.11) | 0.73 (0.63-0.85) |
| Model 3, HR (95% CI) | 1.00 (ref) | 1.20 (0.83-1.73) | 1.30 (1.12-1.50) | 1.05 (0.89-1.24) |
| **2-year mortality** |  |  |  |  |
| Model 1, HR (95% CI) | 1.00 (ref) | 0.59 (0.42-0.84) | 1.17 (1.02-1.34) | 0.67 (0.58-0.78) |
| Model 2, HR (95% CI) | 1.00 (ref) | 0.94 (0.66-1.33) | 0.99 (0.86-1.13) | 0.75 (0.64-0.87) |
| Model 3, HR (95% CI) | 1.00 (ref) | 1.36 (0.95-1.96) | 1.28 (1.11-1.48) | 1.02 (0.86-1.20) |
| **5-year mortality** |  |  |  |  |
| Model 1, HR (95% CI) | 1.00 (ref) | 0.72 (0.48-1.08) | 1.31 (1.11-1.54) | 0.71 (0.59-0.86) |
| Model 2, HR (95% CI) | 1.00 (ref) | 1.15 (0.76-1.73) | 1.11 (0.94-1.31) | 0.82 (0.68-0.98) |
| Model 3, HR (95% CI) | 1.00 (ref) | 1.58 (1.04-2.40) | 1.32 (1.11-1.58) | 0.99 (0.81-1.20) |

Abbreviations: CI, confidence interval; HR, hazard ratio

Model 1: Not adjusted for any covariate

Model 2: Adjusted for age and sex

Model 3: Adjusted for age, sex, race, history of MI/PCI/CABG, smoking status, Killip class on admission, creatinine on admission, haemoglobin on admission, MI type, PCI/CABG during hospitalization, drugs (aspirin, P2Y12 inhibitor, beta blocker, ACEI/ARB, lipid lowering drug) given during hospitalization

Supplementary Table 8. Risk of all-cause mortality in Indian AMI patients by obesity and metabolic health

|  | **Metabolically healthy** | | **Metabolically unhealthy** | |
| --- | --- | --- | --- | --- |
|  | **Non-obese (MHN)** | **Obese**  **(MHO)** | **Non-obese (MUN)** | **Obese**  **(MUO)** |
| **In-hospital mortality** |  |  |  |  |
| Model 1, HR (95% CI) | 1.00 (ref) | 0.72 (0.38-1.35) | 0.78 (0.61-1.01) | 0.59 (0.44-0.78) |
| Model 2, HR (95% CI) | 1.00 (ref) | 0.93 (0.49-1.76) | 0.71 (0.55-0.91) | 0.62 (0.47-0.83) |
| Model 3, HR (95% CI) | 1.00 (ref) | 0.74 (0.38-1.42) | 1.26 (0.95-1.65) | 1.16 (0.85-1.58) |
| **30-day mortality** |  |  |  |  |
| Model 1, HR (95% CI) | 1.00 (ref) | 0.58 (0.31-1.10) | 0.98 (0.77-1.26) | 0.62 (0.47-0.82) |
| Model 2, HR (95% CI) | 1.00 (ref) | 0.91 (0.48-1.72) | 0.78 (0.61-1.00) | 0.64 (0.48-0.85) |
| Model 3, HR (95% CI) | 1.00 (ref) | 0.92 (0.49-1.76) | 1.44 (1.10-1.88) | 1.26 (0.93-1.71) |
| **1-year mortality** |  |  |  |  |
| Model 1, HR (95% CI) | 1.00 (ref) | 0.53 (0.31-0.90) | 1.21 (0.99-1.48) | 0.72 (0.57-0.90) |
| Model 2, HR (95% CI) | 1.00 (ref) | 0.83 (0.48-1.43) | 0.96 (0.78-1.17) | 0.73 (0.58-0.92) |
| Model 3, HR (95% CI) | 1.00 (ref) | 0.97 (0.56-1.69) | 1.42 (1.14-1.77) | 1.14 (0.89-1.46) |
| **2-year mortality** |  |  |  |  |
| Model 1, HR (95% CI) | 1.00 (ref) | 0.65 (0.40-1.08) | 1.32 (1.07-1.62) | 0.79 (0.63-1.00) |
| Model 2, HR (95% CI) | 1.00 (ref) | 1.00 (0.61-1.66) | 1.05 (0.86-1.30) | 0.82 (0.65-1.03) |
| Model 3, HR (95% CI) | 1.00 (ref) | 1.09 (0.66-1.82) | 1.49 (1.19-1.85) | 1.19 (0.93-1.52) |
| **5-year mortality** |  |  |  |  |
| Model 1, HR (95% CI) | 1.00 (ref) | 0.54 (0.27-1.08) | 1.52 (1.18-1.94) | 1.01 (0.78-1.32) |
| Model 2, HR (95% CI) | 1.00 (ref) | 0.82 (0.41-1.64) | 1.31 (1.02-1.67) | 1.12 (0.86-1.47) |
| Model 3, HR (95% CI) | 1.00 (ref) | 0.83 (0.41-1.68) | 1.54 (1.19-2.01) | 1.34 (1.01-1.79) |

Abbreviations: CI, confidence interval; HR, hazard ratio

Model 1: Not adjusted for any covariate

Model 2: Adjusted for age and sex

Model 3: Adjusted for age, sex, race, history of MI/PCI/CABG, smoking status, Killip class on admission, creatinine on admission, haemoglobin on admission, MI type, PCI/CABG during hospitalization, drugs (aspirin, P2Y12 inhibitor, beta blocker, ACEI/ARB, lipid lowering drug) given during hospitalization

Supplementary Table 9. Risk of all-cause mortality in AMI patients who never smoke by obesity and metabolic health

|  | **Metabolically healthy** | | **Metabolically unhealthy** | |
| --- | --- | --- | --- | --- |
|  | **Non-obese (MHN)** | **Obese**  **(MHO)** | **Non-obese (MUN)** | **Obese**  **(MUO)** |
| **In-hospital mortality** |  |  |  |  |
| Model 1, HR (95% CI) | 1.00 (ref) | 0.77 (0.56-1.06) | 0.88 (0.79-0.97) | 0.68 (0.60-0.76) |
| Model 2, HR (95% CI) | 1.00 (ref) | 1.00 (0.72-1.39) | 0.83 (0.75-0.92) | 0.77 (0.69-0.87) |
| Model 3, HR (95% CI) | 1.00 (ref) | 1.10 (0.80-1.53) | 1.41 (1.27-1.56) | 1.45 (1.28-1.63) |
| **30-day mortality** |  |  |  |  |
| Model 1, HR (95% CI) | 1.00 (ref) | 0.57 (0.41-0.79) | 0.89 (0.81-0.98) | 0.58 (0.51-0.65) |
| Model 2, HR (95% CI) | 1.00 (ref) | 0.91 (0.66-1.25) | 0.80 (0.73-0.89) | 0.70 (0.62-0.79) |
| Model 3, HR (95% CI) | 1.00 (ref) | 1.17 (0.85-1.62) | 1.42 (1.29-1.57) | 1.44 (1.28-1.63) |
| **1-year mortality** |  |  |  |  |
| Model 1, HR (95% CI) | 1.00 (ref) | 0.49 (0.38-0.64) | 0.97 (0.90-1.05) | 0.57 (0.52-0.62) |
| Model 2, HR (95% CI) | 1.00 (ref) | 0.80 (0.61-1.04) | 0.88 (0.81-0.94) | 0.70 (0.64-0.76) |
| Model 3, HR (95% CI) | 1.00 (ref) | 1.11 (0.85-1.45) | 1.31 (1.21-1.41) | 1.14 (1.04-1.25) |
| **2-year mortality** |  |  |  |  |
| Model 1, HR (95% CI) | 1.00 (ref) | 0.48 (0.37-0.63) | 1.00 (0.93-1.08) | 0.61 (0.56-0.67) |
| Model 2, HR (95% CI) | 1.00 (ref) | 0.82 (0.63-1.08) | 0.90 (0.84-0.98) | 0.75 (0.69-0.82) |
| Model 3, HR (95% CI) | 1.00 (ref) | 1.13 (0.86-1.48) | 1.30 (1.20-1.41) | 1.15 (1.05-1.27) |
| **5-year mortality** |  |  |  |  |
| Model 1, HR (95% CI) | 1.00 (ref) | 0.48 (0.35-0.67) | 1.02 (0.93-1.12) | 0.64 (0.57-0.71) |
| Model 2, HR (95% CI) | 1.00 (ref) | 0.84 (0.60-1.17) | 0.94 (0.86-1.03) | 0.82 (0.73-0.91) |
| Model 3, HR (95% CI) | 1.00 (ref) | 1.10 (0.79-1.53) | 1.25 (1.13-1.38) | 1.15 (1.03-1.29) |

Abbreviations: CI, confidence interval; HR, hazard ratio

Model 1: Not adjusted for any covariate

Model 2: Adjusted for age and sex

Model 3: Adjusted for age, sex, race, history of MI/PCI/CABG, smoking status, Killip class on admission, creatinine on admission, haemoglobin on admission, MI type, PCI/CABG during hospitalization, drugs (aspirin, P2Y12 inhibitor, beta blocker, ACEI/ARB, lipid lowering drug) given during hospitalization

Supplementary Table 10. Risk of all-cause mortality in AMI patients who were ex-smokers by obesity and metabolic health

|  | **Metabolically healthy** | | **Metabolically unhealthy** | |
| --- | --- | --- | --- | --- |
|  | **Non-obese (MHN)** | **Obese**  **(MHO)** | **Non-obese (MUN)** | **Obese**  **(MUO)** |
| **In-hospital mortality** |  |  |  |  |
| Model 1, HR (95% CI) | 1.00 (ref) | 0.84 (0.50-1.42) | 0.73 (0.63-0.85) | 0..46 (0.37-0.56) |
| Model 2, HR (95% CI) | 1.00 (ref) | 1.15 (0.68-1.95) | 0.73 (0.62-0.85) | 0.58 (0.47-0.70) |
| Model 3, HR (95% CI) | 1.00 (ref) | 1.32 (0.78-2.24) | 1.15 (0.98-1.34) | 0.98 (0.80-1.21) |
| **30-day mortality** |  |  |  |  |
| Model 1, HR (95% CI) | 1.00 (ref) | 0.60 (0.37-0.97) | 0.76 (0.66-0.88) | 0.38 (0.31-0.46) |
| Model 2, HR (95% CI) | 1.00 (ref) | 1.00 (0.62-1.63) | 0.72 (0.62-0.84) | 0.52 (0.43-0.63) |
| Model 3, HR (95% CI) | 1.00 (ref) | 1.25 (0.77-2.04) | 1.17 (0.99-1.36) | 0.97 (0.79-1.19) |
| **1-year mortality** |  |  |  |  |
| Model 1, HR (95% CI) | 1.00 (ref) | 0.38 (0.24-0.59) | 0.85 (0.76-0.95) | 0.42 (0.36-0.48) |
| Model 2, HR (95% CI) | 1.00 (ref) | 0.62 (0.40-0.97) | 0.80 (0.72-0.90) | 0.56 (0.48-0.65) |
| Model 3, HR (95% CI) | 1.00 (ref) | 0.77 (0.49-1.19) | 1.06 (0.95-1.20) | 0.83 (0.72-0.97) |
| **2-year mortality** |  |  |  |  |
| Model 1, HR (95% CI) | 1.00 (ref) | 0.42 (0.28-0.65) | 0.88 (0.79-0.99) | 0.46 (0.40-0.53) |
| Model 2, HR (95% CI) | 1.00 (ref) | 0.66 (0.43-1.02) | 0.83 (0.74-0.93) | 0.61 (0.53-0.71) |
| Model 3, HR (95% CI) | 1.00 (ref) | 0.80 (0.52-1.24) | 1.06 (0.94-1.19) | 0.86 (0.74-1.00) |
| **5-year mortality** |  |  |  |  |
| Model 1, HR (95% CI) | 1.00 (ref) | 0.46 (0.28-0.75) | 0.92 (0.80-1.05) | 0.48 (0.41-0.57) |
| Model 2, HR (95% CI) | 1.00 (ref) | 0.76 (0.46-1.24) | 0.91 (0.79-1.04) | 0.70 (0.59-0.83) |
| Model 3, HR (95% CI) | 1.00 (ref) | 1.10 (0.67-1.81) | 1.04 (0.90-1.20) | 0.83 (0.70-0.99) |

Abbreviations: CI, confidence interval; HR, hazard ratio

Model 1: Not adjusted for any covariate

Model 2: Adjusted for age and sex

Model 3: Adjusted for age, sex, race, history of MI/PCI/CABG, smoking status, Killip class on admission, creatinine on admission, haemoglobin on admission, MI type, PCI/CABG during hospitalization, drugs (aspirin, P2Y12 inhibitor, beta blocker, ACEI/ARB, lipid lowering drug) given during hospitalization

Supplementary Table 11. Risk of all-cause mortality in AMI patients who were current smokers by obesity and metabolic health

|  | **Metabolically healthy** | | **Metabolically unhealthy** | |
| --- | --- | --- | --- | --- |
|  | **Non-obese (MHN)** | **Obese**  **(MHO)** | **Non-obese (MUN)** | **Obese**  **(MUO)** |
| **In-hospital mortality** |  |  |  |  |
| Model 1, HR (95% CI) | 1.00 (ref) | 0.75 (0.48-1.16) | 0.76 (0.65-0.88) | 0.44 (0.35-0.55) |
| Model 2, HR (95% CI) | 1.00 (ref) | 1.02 (0.66-1.59) | 0.72 (0.62-0.84) | 0.56 (0.45-0.71) |
| Model 3, HR (95% CI) | 1.00 (ref) | 0.72 (0.46-1.13) | 1.18 (1.00-1.38) | 1.00 (0.79-1.27) |
| **30-day mortality** |  |  |  |  |
| Model 1, HR (95% CI) | 1.00 (ref) | 0.52 (0.34-0.80) | 0.85 (0.73-0.98) | 0.38 (0.31-0.48) |
| Model 2, HR (95% CI) | 1.00 (ref) | 0.86 (0.56-1.32) | 0.75 (0.65-0.87) | 0.55 (0.44-0.68) |
| Model 3, HR (95% CI) | 1.00 (ref) | 0.86 (0.56-1.32) | 1.11 (0.95-1.30) | 0.88 (0.70-1.10) |
| **1-year mortality** |  |  |  |  |
| Model 1, HR (95% CI) | 1.00 (ref) | 0.51 (0.36-0.72) | 1.00 (0.89-1.12) | 0.48 (0.41-0.57) |
| Model 2, HR (95% CI) | 1.00 (ref) | 0.86 (0.61-1.22) | 0.87 (0.78-0.98) | 0.69 (0.59-0.82) |
| Model 3, HR (95% CI) | 1.00 (ref) | 0.86 (0.61-1.22) | 1.04 (0.92-1.17) | 0.89 (0.75-1.05) |
| **2-year mortality** |  |  |  |  |
| Model 1, HR (95% CI) | 1.00 (ref) | 0.55 (0.39-0.77) | 1.03 (0.92-1.16) | 0.51 (0.43-0.60) |
| Model 2, HR (95% CI) | 1.00 (ref) | 0.91 (0.65-1.27) | 0.90 (0.80-1.01) | 0.72 (0.61-0.85) |
| Model 3, HR (95% CI) | 1.00 (ref) | 0.85 (0.61-1.19) | 1.00 (0.89-1.13) | 0.83 (0.71-0.98) |
| **5-year mortality** |  |  |  |  |
| Model 1, HR (95% CI) | 1.00 (ref) | 0.55 (0.37-0.83) | 1.16 (1.02-1.32) | 0.59 (0.50-0.71) |
| Model 2, HR (95% CI) | 1.00 (ref) | 0.85 (0.57-1.27) | 1.03 (0.90-1.17) | 0.84 (0.70-1.01) |
| Model 3, HR (95% CI) | 1.00 (ref) | 0.69 (0.46-1.04) | 0.94 (0.82-1.08) | 0.79 (0.66-0.95) |

Abbreviations: CI, confidence interval; HR, hazard ratio

Model 1: Not adjusted for any covariate

Model 2: Adjusted for age and sex

Model 3: Adjusted for age, sex, race, history of MI/PCI/CABG, smoking status, Killip class on admission, creatinine on admission, haemoglobin on admission, MI type, PCI/CABG during hospitalization, drugs (aspirin, P2Y12 inhibitor, beta blocker, ACEI/ARB, lipid lowering drug) given during hospitalization
